# Anti-Breast Cancer Activity of Latroeggtoxin-V Mined from the Transcriptome of Spider *Latrodectus tredecimguttatus* Eggs

**DOI:** 10.3390/toxins10110451

**Published:** 2018-11-02

**Authors:** Dehong Xu, Xiaochao Tang, Xinzhou Wu, Dianmei Yu, Pingping Tang, Xianchun Wang

**Affiliations:** 1 Key Laboratory of Protein Chemistry and Developmental Biology of Ministry of Education, College of Life Sciences, Hunan Normal University, Changsha 410081, China; xudehong163@126.com (D.X.); flower92931@outlook.com (X.T.); wuxinzhouwu@163.com (X.W.); yudianmei@outlook.com (D.Y.); Pingpingtang@smail.hunnu.edu.cn (P.T.); 2 Laboratory of Biological Engineering, College of Pharmacy, Hunan University of Chinese Medicine, Changsha 410208, China

**Keywords:** Latroeggtoxin-V, Recombinant expression, MDA-MB-231, anticancer activity, ATPase inhibitor, *Latrodectus tredecimguttatus* egg

## Abstract

As a black widow spider, *Latrodectus tredecimguttatus* has poisonous components not only in venomous glands but also in eggs. Our previous work had carried out a transcriptome analysis of the spider eggs in an attempt to probe into the molecular basis of the egg toxicity. A proteinaceous toxin, named Latroeggtoxin-V, was mined from the identified transcriptome. In this study, the gene of Latroeggtoxin-V was cloned and heterologously expressed, and the anticancer activity of the recombinant Latroeggtoxin-V (rLatroeggtoxin-V) was characterized. Activity assay found that rLatroeggtoxin-V could selectively act on breast cancer line MDA-MB-231 cells, not only arresting their cell cycle, inhibiting their proliferation and migration, but also inducing their apoptosis. Bioinformatics analysis suggested that Latroeggtoxin-V belongs to the ATPase inhibitor protein family and the further activity assay showed that the rLatroeggtoxin-V inhibited the activity of the Na^+^/K^+^-ATPase in MDA-MB-231 cells in a concentration-dependent manner, suggesting that the anticancer activity of Latroeggtoxin-V is based on its affecting the ion transport and receptor functions of Na^+^/K^+^-ATPase. The present work not only laid the foundation for the utilization of Latroeggtoxin-V in the anticancer drug development and the related fields, but also provided a new paradigm for exploration of the proteinaceous toxins under the direction of transcriptomics and bioinformatics.

## 1. Introduction

Spider *Latrodectus tredecimguttatus*, also known as the “black widow”, belongs to *Arthropoda*, *Arachnoidea*, *Araneida*, *Theridiidae*, *Latrodectus* in zoology and is one of the most poisonous spiders known in the world [1,2]. *L. tredecimguttatus* can release highly toxic venom, causing severe pain in the whole body after being stung by it and leading to functional or organic diseases of single or multiple organs such as liver, brain, kidney, heart and lung, and can even lead to death [3,4]. Furthermore, it had been found that not only does the venom of *L. tredecimguttatus* contain many toxic components [5,6,7,8,9], but that other parts of the body and even the eggs produced by it are also toxic [5,10,11,12]. In recent years, our research group has carried out a systematic study on the toxicity of the eggs using a combination of multiple techniques including proteomics and transcriptomics. The proteomic results showed that there are a variety of proteinaceous toxins in the eggs, which are significantly different from those in the venom, indicating that the eggs have the distinct molecular basis of toxicity [13]. By comprehensively using multiple techniques, four proteinaceous toxins, named Latroeggtoxin-I to Latroeggtoxin-IV, were purified and characterized from the eggs. Latroegtoxin-I is a neurotoxic protein and can block the neuromuscular transmission [14]. Latroeggtoxin-II selectively inhibits the TTX-R Na^+^ channel current in rat dorsal root ganglion neurons, showing toxicity toward both mice and *Periplaneta americana* [15]. Latroeggtoxin-III is an insect-specific protein toxin, whereas Latroeggtoxin-IV an antibacterial peptide [16]. No doubt, these proteinaceous toxins play important roles in the egg toxicity. However, there must be other active components that participate in the egg toxicity. Nevertheless, some of them, due to their too low abundance, are difficult to purify from the eggs, which limits us to understanding the molecular basis of the egg toxicity and utilizing the active components in the eggs.

At present, various omics strategies have successively emerged in the field of life science, of which transcriptomics based on the second generation high-throughput sequencing has been widely used in gene expression analysis. With this technique, the researchers can comprehensively and rapidly obtain the genomic transcription information of the researched object, which is helpful for revealing the molecular basis and mechanism underlying different biological characteristics [17,18]. Our group has carried out a transcriptomic analysis of *L. tredecimguttatus* eggs, from which 280 open reading frames encoding possible proteinaceous toxins were identified and the biological functions of the encoded toxins were bioinformatically predicted [19], thus providing guidance for the subsequent gene cloning and activity screening. One open reading frames has attracted our attention because the protein it encodes has high homology with the reported anticancer peptide SK84 [20], suggesting that the egg protein might also have anticancer activity. Our present study cloned and heterologously expressed the gene of the egg protein, and experimentally demonstrated that this protein, named Latroeggtoxin-V, is an ATPase inhibitor and has anticancer properties toward breast cancer line MDA-MB-231 cells, exhibiting potential application in the development of anticancer drugs.

## 2. Results

### 2.1. Bioinformatic Analysis on Latroeggtoxin-V

**Figure 1:**
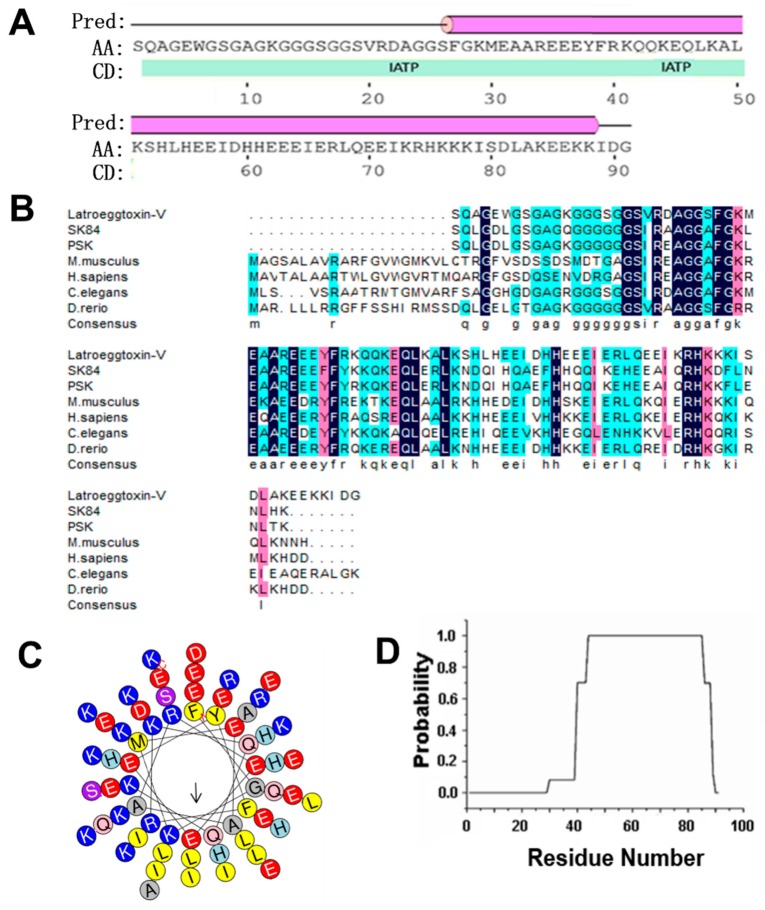
Bioinformatics analysis of Latroeggtoxin-V. (**A**) The conserved domain and secondary structures of Latroeggtoxin-V. The ‘pred’ represents predicted secondary structure, of which the line represents coil, and the cylinder represents α-helix. ‘AA’, ‘CD’ and ‘IATP’ indicate query sequence (Latroeggtoxin-V), conserved domain and ATPase inhibitor domain, respectively. (**B**) Homologous sequence alignment of Latroeggtoxin-V. Latroeggtoxin-V was aligned with mitochondrial ATPase inhibitory peptides from Drosophila melanogaster (SK84 and PSK), Mus musculus (NP_031538.2), Homo sapiens (NP_057395.1), Caenorhabditis elegans (NP_500336.1) and Danio rerio (NP_001082990.1). The identical residues are shaded in deep blue. The solid line at the bottom indicates the fragment corresponding to the minimal inhibitory sequence in ATPase inhibitor of bovine. (**C**) The helical wheel plots of α-helix in Latroeggtoxin-V. The α-helix of Latroeggtoxin-V shows amphipathic structural features, of which the hydrophobic face is indicated by an arrow. (**D**) Prediction of α-helical coiled-coil in Latroeggtoxin-V. The line represents the probability of forming α-helical coiled-coil participated in by each residue in Latroeggtoxin-V, where the C terminal residues from K44 to E85 have the maximal probability (100%) to form α-helical coiled-coil.

Bioinformatic analysis on Latroeggtoxin-V showed that the theoretical molecular weight (MW) and isoelectric point (pI) of this protein were 10.17 kDa and 6.21, respectively. The prediction of secondary structure indicated that two kinds of secondary structure units were contained in the Latroeggtoxin-V molecule: α-helix and coil. The secondary structure was dominated by α-helix formed by C-terminal sequence, accounting for 68.1% of the total sequence, while the remaining 21.9% was in the coil conformation (Figure 1A). The search of conserved domain found that Latroeggtoxin-V has ATPase inhibitor domain and its sequence was homologous with mitochondrial ATPase inhibitors from several different organisms (Figure 1A,B), so it was speculated that Latroeggtoxin-V belongs to the mitochondrial ATPase inhibitor family. Of the homologous ATPase inhihtors, Sk84 and PSK (a peptide with terminal S and K residues) were reported to have anticancer and antibacterial activities [20,21], suggesting that Latroeggtoxin-V may have such bioactivities. Besides, by analyzing the hydrophobicity of α-helix in Latroeggtoxin-V, it was found that the α-helix at the C-terminus of Latroeggtoxin-V was amphipathic, with more hydrophobic amino acid residues, such as L, I, and F, being distributed on one side and more hydrophillic amino acid residues, such as K, E, D, and R, on the other (Figure 1C). In addition, COIL Server analysis showed that the α-helix in C-terminal sequence of Latroeggtoxin-V could form α-helical coiled-coil conformation (Figure 1D), suggesting that Latroeggtoxin-V was likely to dimerize with itself through this structure [22].

### 2.2. Gene Cloning of Latroeggtoxin-V

**Figure 2:**
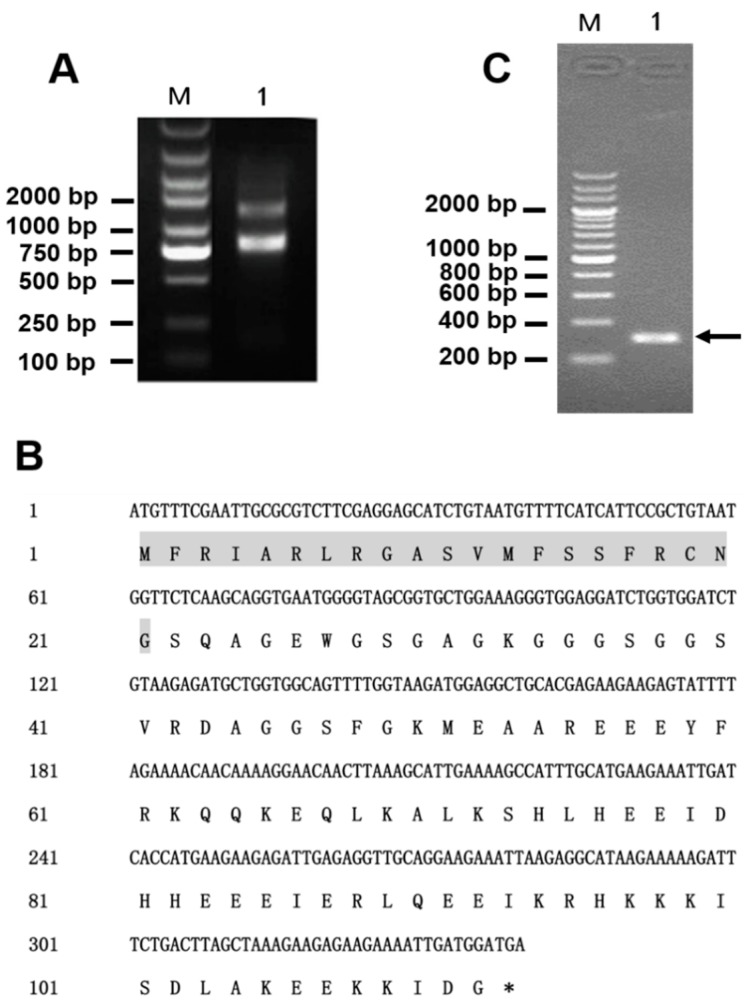
Gene cloning of Latroeggtoxin-V. (**A**) Identification of the total RNA integrity with agarose gel electrophoresis. M, DNA marker; Lane 1, total RNA. (**B**) Nucleic acid sequence encoding Latroeggtoxin-V and the corresponding amino acid sequence. The signal sequence is shaded in gray, followed by the mature peptide sequence of Latingtoxin-V. The asterisk indicates the stop codon. (**C**) Detection of PCR product of Latroeggtoxin-V gene with agarose gel electrophoresis. M, DNA marker; Lane 1, PCR product, a 310-bp nucleic acid sequence containing the 276-bp gene fragment and restriction endonuclease and enterokinase cleavage site sequences.

The extracted total RNA had excellent integrity confirmed by agarose gel electrophoresis (Figure 2A) and was of high purity (OD_260 nm_/OD_280 nm_ = 1.9), with a concentration of 900 μg/mL. According to the results of previous transcriptomic analysis of the eggs of *L. tredecimguttatus*, the nucleic acid sequence encoding Latroeggtoxin-V as well as amino acid sequence was obtained (Figure 2B). Based on the sequence, gene-specific primers (*Latroeggtoxin-V*-F and *Latroeggtoxin-V*-R) were designed and used for PCR reaction, using the cDNA prepared by reverse transcription from the extracted total RNA as the template. Agarose gel electrophoresis and sequencing analysis showed that the PCR product was a 310-bp gene fragment that was consistent with that predicted (Figure 2C), suggesting successful gene cloning.

### 2.3. Prokaryotic Expression and Purification of Latroeggtoxin-V Fusion Protein

**Figure 3:**
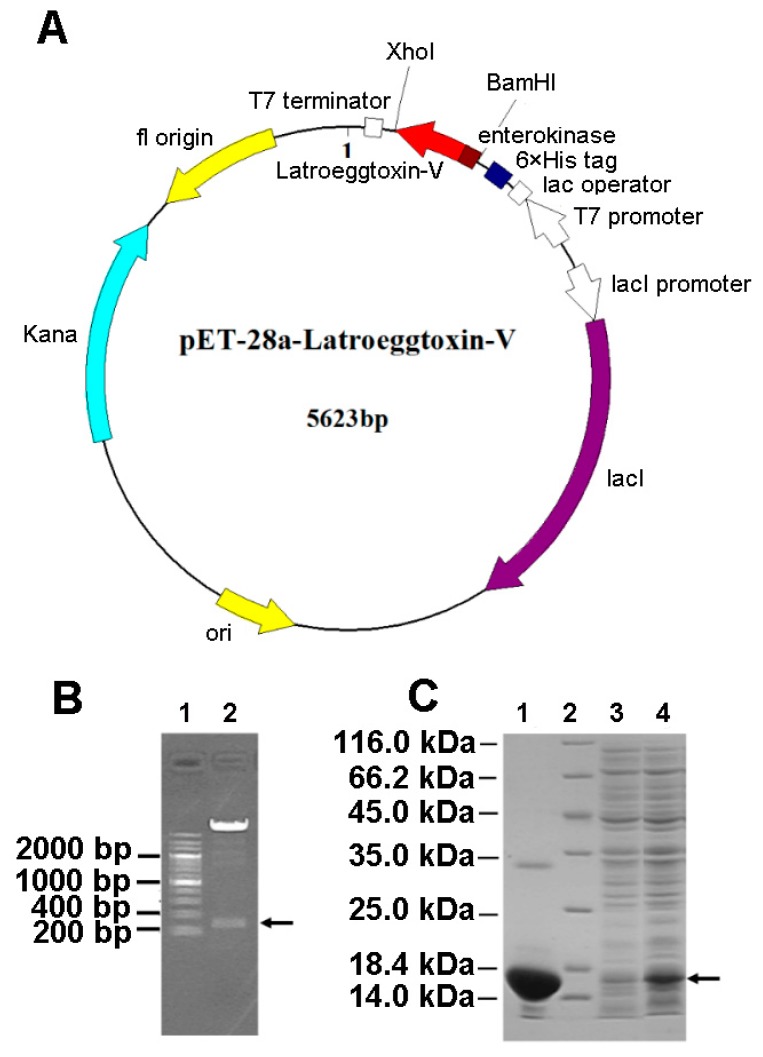
Gene cloning and fusion expression of Latroeggtoxin-V. (**A**) Plasmid map of Latroeggtoxin-V expression vector. The *Latroeggtoxin-V* gene fragment was cloned into the expression vector pET-28a and expressed in the form of fusion protein containing a His tag and enterokinase cleavage sites. (**B**) Identification of recombinant plasmid pET-28a-*Latroeggtoxin-V* by double enzyme digestion. Line 1, DNA marker; Lane 2, products of double enzyme digestion by BamHI and XhoI. The arrow indicates the *Latroeggtoxin-V* gene fragment. (**C**) SDS-PAGE image showing expression and purification of Latroeggtoxin-V fusion protein. Line 2, protein marker; Lanes 1, 3 and 4, Latroeggtoxin-V fusion protein after purification, cell lysate before inducing by IPTG, and cell lysate after inducing by IPTG for 5 h, respectively. The arrow indicates the expressed fusion protein.

The gene fragment encoding the Latroeggtoxin-V mature peptide was successfully inserted between BamHI and XhoI sites in the pET-28a expression vector (Figure 3A), confirmed by double enzyme digestion (Figure 3B). After the recombinant vector was transformed into BL21 (DE3) *E. coli*, the protein expression was induced with IPTG, and SDS-PAGE showed that *Latroeggtoxin-V* gene was efficiently expressed in the form of fusion protein with a molecular weight of about 14.30 kDa consistent with that predicted (Figure 3C). After the *E. coli* cells were collected, lysed by sonication and centrifuged, the fusion protein in the supernatant was purified in a batchwise manner by Ni-NTA affinity chromatography under native conditions. The target fusion protein was eluted with 250 mM imidazole, and SDS-PAGE demonstrated that the fusion protein had been efficiently purified (Figure 3C).

### 2.4. Acquisition and Identification of rLatroeggtoxin-V

**Figure 4:**
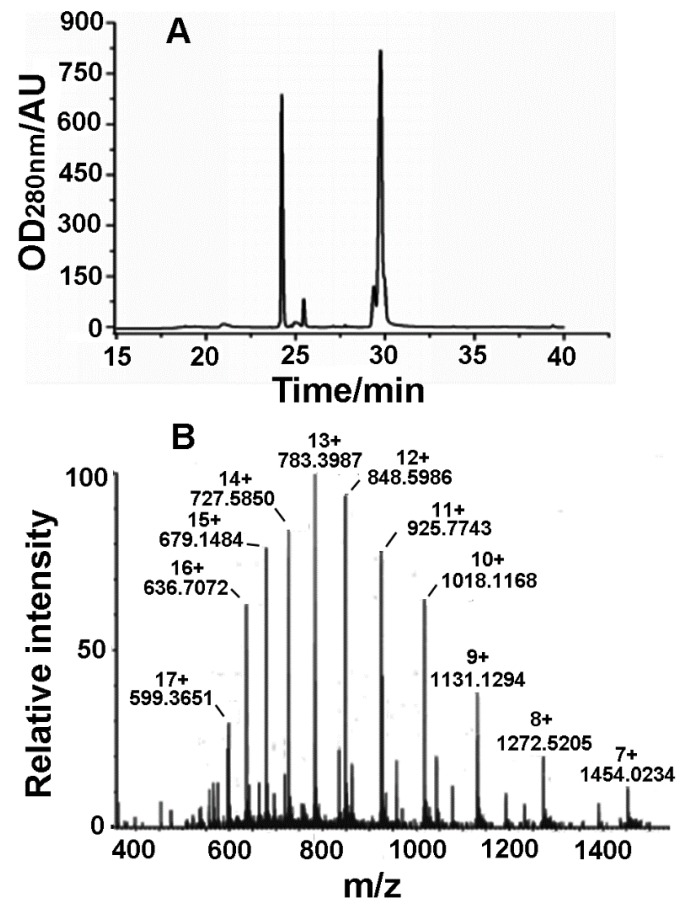
Purification and identification of rLatroeggtoxin-V. (**A**) Chromatogram of RP-HPLC purification of rLatroeggtoxin-V. (**B**) Molecular weight determination of rLatroeggtoxin-V by electrospray ionization mass spectrometry. The *m*/*z* value and the number of charges for each rLatroeggtoxin-V ion peak were labeled.

Since the Latroeggtoxin-V fusion protein contains an enterokinase cleavage site, the fusion protein tag can be removed with enterokinase. The products resulted from the enzymatic cleavage were separated by RP-HPLC (Figure 4A). From the figure, two main peaks were observed, and the molecular weight of the protein in the second peak at about 29.8 min was 10.1722 kDa identified by electrospray mass spectrometry (Figure 4B), which was consistent with the theoretical molecular weight of Latroeggtoxin-V, so that the peak was determined to be the desired peak. In addition, the mass spectrum shows a group of regular multiple-charge peaks, indicating that the rLatroeggtoxin-V sample had been purified.

### 2.5. Effects of rLatroeggtoxin-V on Cell Viability

**Figure 5:**
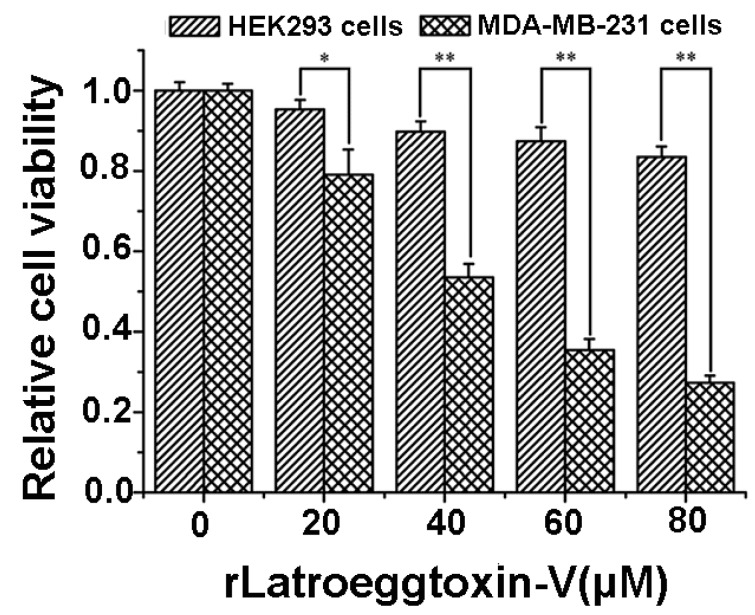
Comparison of the inhibitory effects of rLatroeggtoxin-V on the proliferation in HEK293 and MDA-MB-231 cells. * *p* < 0.05; ** *p* < 0.01. The data are represented as mean ± SD. (*n* = 4).

After the MDA-MB-231 and HEK293 cells were treated with different concentrations of rLatroeggtoxin-V (0, 20, 40, 60 and 80 μM) for 48 h, the cell viability was assessed by MTT assay. The results (Figure 5) showed that rLatroeggtoxin-V decreased the cell viability both of the MDA-MB-231 and HEK293 cells in a concentration-dependent fashion, suggesting that rLatroeggtoxin-V inhibited the proliferation of the two kinds of cells. However, the inhibition of proliferation in MDA-MB-231 cells by rLatroeggtoxin-V was much more significant than that in HEK293 cells (*p* < 0.05 and 0.01), indicating that the inhibitory effect of rLatroeggtoxin-V on the proliferation in MDA-MB-231 breast cancer cells has selectivity. The IC_50_ of rLatroeggtoxin-V against MDA-MB-231 cells was calculated to be about 43 μM.

### 2.6. Apoptosis of MDA-MB-231 Cells Induced by rLatroeggtoxin-V

**Figure 6:**
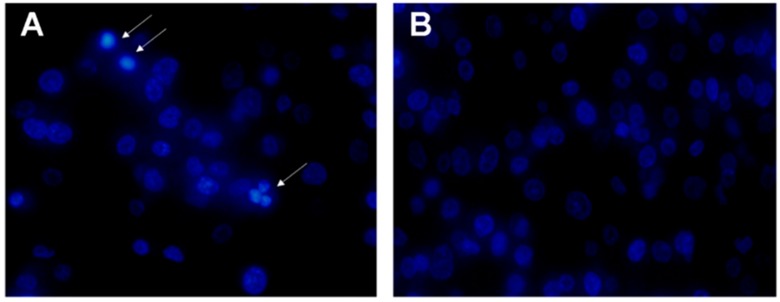
Hoechst 33258 staining for observing apoptosis of MDA-MB-231 cells induced by rLatroeggtoxin-V. (**A**) The fluorescence micrograph of 40 μM rLatroeggtoxin-V treatment for 48 h (400×). White arrows indicate apoptotic cells with pyknotic nucleus. (**B**) The fluorescence micrograph of untreated MDA-MB-231 cells (400×).

In order to investigate whether rLatroeggtoxin-V, besides inhibiting the proliferation in the MDA-MB-231 cells, can decrease the viability of MDA-MB-231 cells through inducing apoptosis, apoptotic morphology of the cells treated with 40 μM rLatroeggtoxin-V and untreated cells was comparatively observed after Hoechst 33258 staining. The results showed that the karyopyknosis characteristic of apoptosis appeared in some cells treated with rLatroeggtoxin-V for 48 h (Figure 6A), but not in untreated cells (Figure 6B). This phenomenon demonstrated that the inhibitory effect of rLatroeggtoxin-V on the viability of MDA-MB-231 cells was partially due to its inducing apoptosis.

### 2.7. Inhibition of MDA-MB-231 Cell Migration by rLatroeggtoxin-V

**Figure 7:**
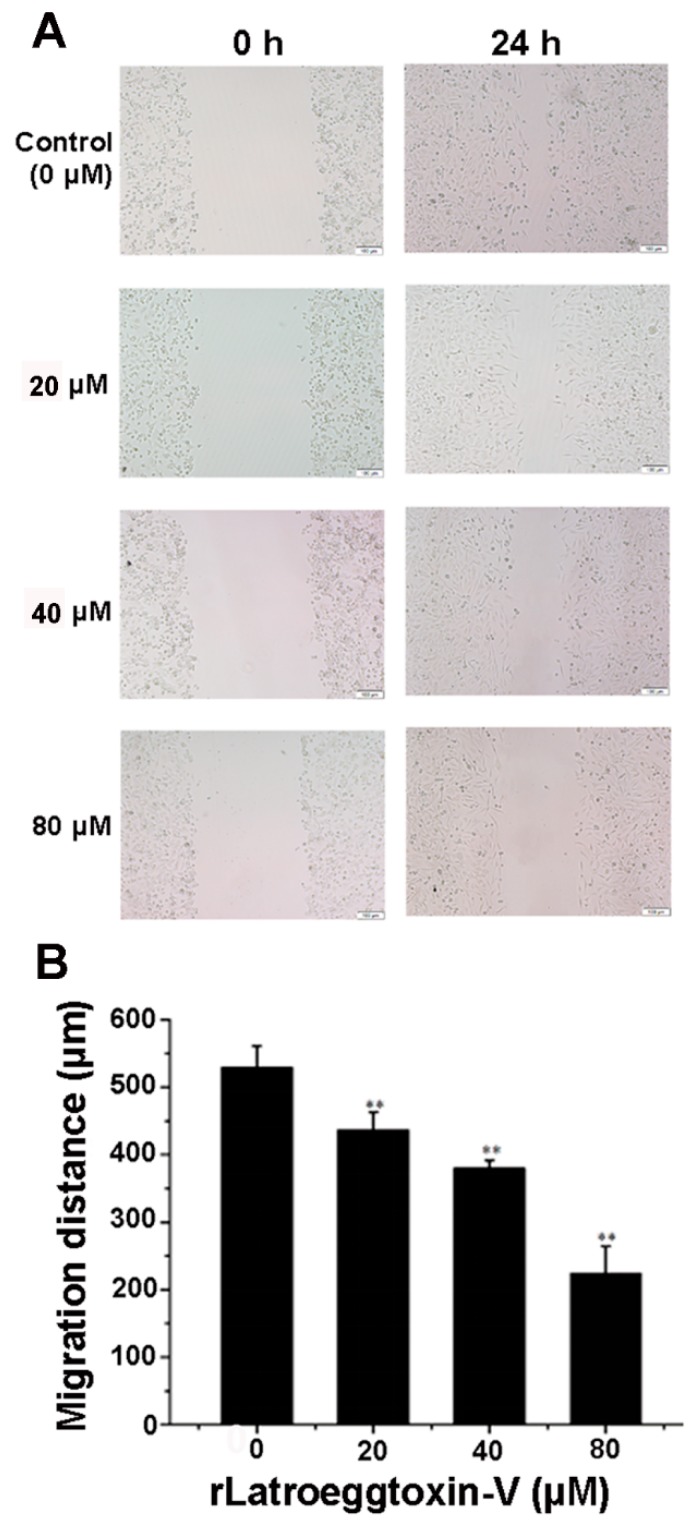
Wound-healing assay for detecting the effect of rLatroeggtoxin-V on MDA-MB-231 cell migration. (**A**) Wound-healing of MDA-MB-231 cells with or without treatment with rLatroeggtoxin-V at different concentrations for 24 h. (**B**) Comparison of MDA-MB-231 cell migration distances after treatment with rLatroeggtoxin-V at different concentrations for 24 h. ** *p* < 0.01 when compared with the control. The data are represented as mean ± SD. (*n* = 3).

In view of the fact that MDA-MB-231 cells have strong invasiveness, the wound-healing assay was performed to detect the effect of rLatroeggtoxin-V on the migration ability of MDA-MB-231 cells in culture. The results demonstrated that the migration distance of MDA-MB-231 cells was continuously decreased as the concentration of the rLatroeggtoxin-V was increased. Compared with the control, the differences reached extremely significant level (*p* < 0.01) (Figure 7). This result demonstrated that rLatroeggtoxin-V could weaken the migration ability of MDA-MB-231 cells in a concentration-dependent manner.

### 2.8. MDA-MB-231 Cell Cycle Arrest Caused by rLatroeggtoxin-V

**Figure 8:**
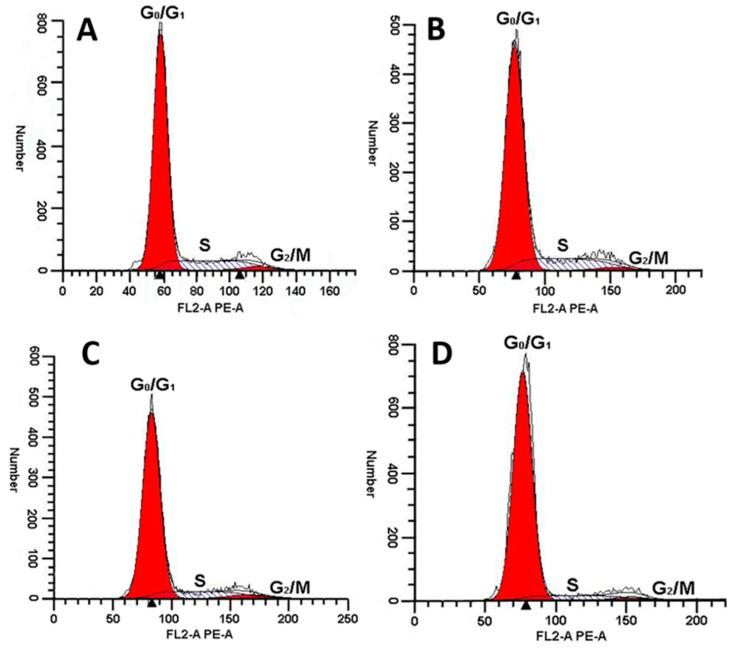
Effect of rLatroeggtoxin-V at different concentrations on MDA-MB-231 cell cycle. Exposure of MDA-MB-231 cells to (**A**, Control) 0 μM, (**B**) 25 μM, (**C**) 50 μM and (**D**) 100 μM rLatroeggtoxin-V for 48 h increased the cell population in the G_0_/G_1_ phases, and decreased that in the S phase.

As shown in Figure 8 and Table 1, rLatroeggtoxin-V could affect the cell cycle distribution of MDA-MB-231. The percentage of MDA-MB-231 cells in G_0_/G_1_ phase increased with the increase of rLatroeggtoxin-V concentration. When the concentrations of rLatroeggtoxin-V were 50 μM and above, the percentage of MDA-MB-231 cells in G_0_/G_1_ phase was significantly higher than that of the control (*p* < 0.05). Correspondingly, the percentage of the MDA-MB-231 cells in S phase was significantly lowed, and that of the MDA-MB-231 cells in G_2_/M phase was not significantly changed. These results indicate that rLatroeggtoxin-V could induce cell cycle arrest at G_0_/G_1_ phase in the MDA-MB-231 cells.

### 2.9. rLatroeggtoxin-V Inhibition of Na^+^/K^+^-ATPase of MDA-MB-231 Cells

**Figure 9:**
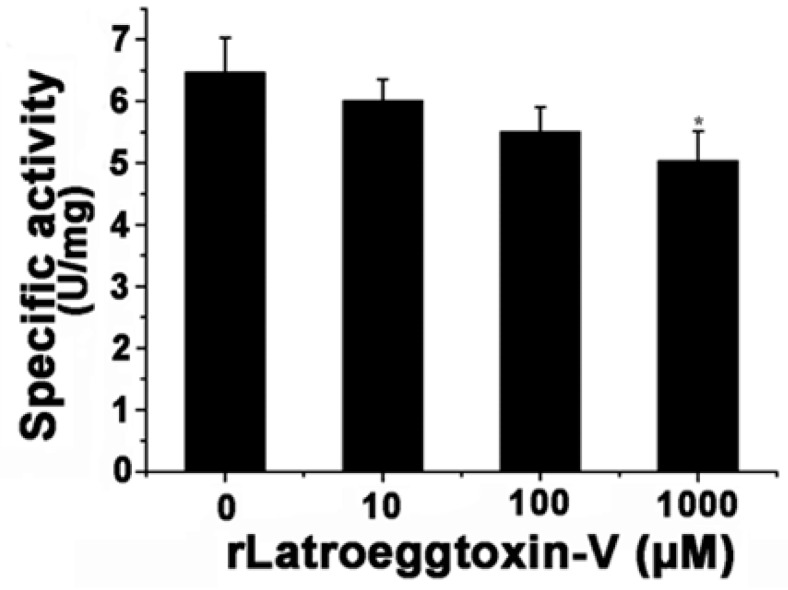
Effect of rLatroeggtoxin-V on the activity of Na^+^/K^+^-ATPase from MDA-MB-231 cells after treatment with different concentrations of rLatroeggtoxin-V. * *p* < 0.05 when compared with the control. The data are represented as mean ± SD. (*n* = 3).

After the MDA-MB-231 cells were treated with different concentrations of rLatroeggtoxin-V (0, 10, 100 and 1000 μM) for 0 and 24 h, the cells were separately harvested and their Na^+^/K^+^-ATPase activities were determined using a Na^+^/K^+^-ATPase assay kit (Solarbio, Shanghai, China). The results showed that rLatroeggtoxin-V could in vitro inhibit the activity of Na^+^/K^+^-ATPase in a concentration-dependent manner (Figure 9). When rLatroeggtoxin-V concentration was 1 mM, the specific activity of the Na^+^/K^+^-ATPase was 5.03 ± 0.48 U/mg, significantly lower than that (6.46 ± 0.57 U/mg) of control (*p* < 0.05).

## 3. Discussion

The conventional strategy for obtaining and studying the desired proteinaceous toxins and other bioactive components is to directly extract them from natural materials followed by activity screening. This strategy has limitations such as high cost, low yield and long period, particularly when the natural material is limited, the material composition is complex, and the desired component is low-abundant. Next-generation sequencing-based transcriptomics provides an effective means to characterize the gene expression products. Theoretically, it is now possible to have virtually all the mRNAs in a sample to be identified and analyzed, which is suitable for rapidly obtaining the inventory of all proteinaceous components without the need for a reference genome. Transcriptome provides much information on the full-length as well as partial gene sequences encoding proteins and peptides, including those expressed in such a low abundance that it is impossible for them to be directly purified from the natural materials. The contemporary bioinformatics may predict the potential biofunctions of the expressed products, and thus narrow the range of activity screening. In our present study, we cloned and heterologously expressed a unigene selected from the *L. tredecimguttatus* egg transcriptome that encodes a protein: Latroeggtoxin-V that had not been isolated from the eggs due to its too low abundance and the complexity of the egg protein composition. BLAST analysis suggested that Latroeggtoxin-V might have activities of inhibiting Na^+^/K^+^-ATPase, anticancer and antibacterium (Figure 1). The experimental results demonstrated that rLatroeggtoxin-V, although showing no antibacterial activity against the tested bacteria *Staphylococcus aureus* and *Bacillus subtilis* (data not shown), could inhibit the Na^+^/K^+^-ATPase, proliferation and migration of breast cancer cell line MDA-MB-231 cells, and induce their apoptosis.

The Na^+^/K^+^-ATPase is a multiple-transmembrane plasma membrane protein complex that utilizes the energy from the hydrolysis of ATP to drive 3 Na^+^ out of cells and 2 K^+^ into cells, which is essential for normal resting membrane potential and maintaining the electrolyte and fluid balance in cells, organs and whole body [23,24,25]. Inhibition of the Na^+^/K^+^-ATPase leads to depletion of intracellular K^+^ and accumulation of intracellular Na^+^, which in turn results in membrane depolarization and increase in cytosolic free Ca^2+^ concentration due to activation of voltage-gated Ca^2+^ channels and reversed operation of the Na^+^/Ca^2+^ exchanger [25,26,27]. Calcium is a highly versatile intracellular signing and regulates many different cellular processes, such as gene transcription, cell growth, cell proliferation, cell differentiation, cell migration, mitochondrial redox state, etc. [28]. In addition, Na^+^/K^+^-ATPase also works as a cell surface receptor and a key player of cell adhesion, participating in modulation of cell signaling mechanism, affecting proliferation, cell–cell interaction, differentiation and apoptosis, etc. [29,30,31]. Generally speaking, Na^+^/K^+^-ATPase is a house-keeping enzyme with multiple specialized functions and is required for the normal physiological and biochemical activities even the survival of all cells [25]. The aberrant expression and activity of the Na^+^/K^+^-ATPase are related to the development and progression of breast cancer, the most prevalent malignancies found in women all over the world [31]. In view of the unique properties of the Na^+^/K^+^-ATPase, this enzyme could be used as a potentially important target for the development of anti-breast cancer drugs. Furthermore, the Na^+^/K^+^-ATPase can serve as a target for a number of ligands, thus providing even more possibilities for screening specific anti-breast cancer drugs [24,31,32,33,34]. For example, there have been reports indicating that cardiac glycosides are potent inhibitors of Na^+^/K^+^-ATPase and possess potent anti-breast cancer activity, displaying potential in the development of anti-breast cancer drugs [31]. Ouabain and digitoxin were demonstrated to be able to efficiently inhibit the migration of breast cancer MDA-MB-231 cells and their antimigratory effects are directly related to the inhibition of Na^+^/K^+^-ATPase-mediated Na^+^/K^+^ transport [35]. Kometiani et al. reported that low concentrations of ouabain (100 nM or lower) caused only <25% inhibition of pumping function of Na^+^/K^+^-ATPase in human breast cancer line MDA-MB-435s, and had no effect on cell viability, but inhibited proliferation by activating the Na^+^/K^+^-ATPase-mediated signaling pathways that led to the increase in the level of cell cycle inhibitor and the growth arrest [36]. However, due to the low (or lack of) specificity and other limitations (such as more easily to produce drug resistance) of the existing anti-cancer agents particularly those of small molecular weight, their clinical applications often have side effects and a relatively low success rate [37,38,39,40], which instigates the need for more effective and less side effect-causing drugs from natural resources.

The proteins and peptides with anticancer activity from the venomous animals constitute a new kind of library for anticancer drug screening. Compared with the small molecular drugs, proteinaceous ones have the advantages such as high selectivity and affinity for their molecular targets and difficultly making tumors develop resistance [41,42]. Until now, several proteinaceous components showing anticancer activities against breast cancer cells have been screened from the venomous animals. For example, melittin, the major peptide component in the venom of honey bee *Apis mellifera*, has been shown to suppress EGF-induced cell motility and invasion by inhibiting PI3K/Akt/mTOR signaling pathway in breast cancer cells [43]. Neopladine 1 and neopladine 2, two novel proteins with molecular weights of 29.918 and 30.388 kDa, respectively, were purified from the venom of scorpion *Tityus discrepans* and found to induce apoptosis of human breast carcinoma SKBR3 cells but has a negligible effect on non-malignant MA104 monkey kidney cells [44]. The proteinaceous toxin C13S1C1 from the venom of Eastern green mamba, *Dendroaspis angusticeps* (Elapidae) shows strong cytotoxicity against breast adenocarcinoma MDA-MB-231 cells [45]. Latroeggtoxin-V investigated in our present study is a proteinaceous toxin mined from the spider *L. tredecimguttatus* egg transcriptomic data with the help of bioinformatics. The experimental results showed that the rLatroeggtoxin-V at the lower tested concentrations could induce apoptosis of breast cancer MDA-MB-231 cells (Figure 6), inhibit migration of the cancer cells, arrest the cell cycle and thus inhibit the proliferation in a concentration-dependent fashion (Figure 5, Figure 7 and Figure 8; Table 1). At the same time, rLatroeggtoxin-V was demonstrated to in vitro inhibit the activity of Na^+^/K^+^-ATPase in MDA-MB-231 cells in a concentration-dependent manner, with the inhibition reaching significant level (*p* < 0.05) at a concentration of 1 mM (Figure 7). It is worthy of mentioning that rLatroeggtoxin-V efficiently inhibit MDA-MB-231 cells proliferation, and, however, the same dose of rLatroeggtoxin-V had much lesser toxic effect on non-tumor- derived HEK 293 cells. The selective action of Latroeggtoxin-V on the breast cancer cells makes the active protein present a promising prospect for the development of related drugs. In addition, the selective action of Latroeggtoxin-V suggests that there are differences in the structures of Latroeggtoxin-V-binding sites in the Na^+^/K^+^-ATPase of cancer cells and normal cells. Obviously, further research of the structural differences would be helpful for elucidating the molecular basis underlying the selectivity of action of Latroeggtoxin-V.

**Figure 10:**
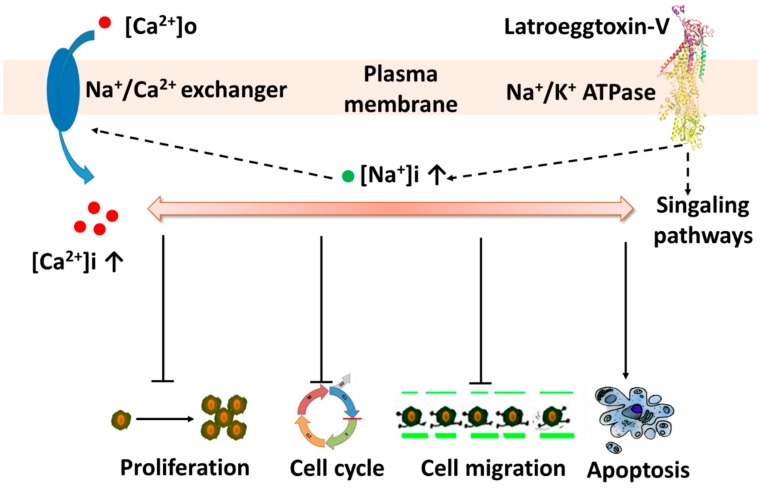
Diagram showing the putative actions of Latroeggtoxin-V on MDA-MB-231 cells through interaction with Na^+^/K^+^-ATPase.

In light of the fact that the Na^+^/K^+^-ATPase not only has an ion transport function, but also has a cell-surface receptor function [29,30], it can be postulated that the Latroeggtoxin-V exerts its effects on the breast cancer cells via at least two ways: Affecting the Na^+^/K^+^-ATPase receptor-mediated signaling pathways and changing the ionic homeostasis by inhibiting the activity of Na^+^/K^+^-ATPase in the MDA-MB-231 cells. Furthermore, although these two action pathways interacted and overlapped with each other to a certain degree, in the lower concentrations the effects of Latroeggtoxin-V on the cancer cells might be due mainly to activation or inhibition of the related signaling pathways, and in the higher concentrations mainly to the disruption of ionic homeostasis, including the depletion of intracellular K^+^, accumulation of Na^+^ and thus the increase of free Ca^2+^ in cytosol [25,27]. Bioinformatic analysis suggested that the C-terminal sequence of Latroeggtoxin-V forms an α-helix with amphipathic structural features, and the residues from K44 to E85 in the C-terminal sequence have the maximal probability (100%) to form α-helical coiled-coil structure by dimerization, which is a prerequisite for the ATPase inhibitory action and biomedical application [46,47,48]. Taken together, the actions of Latroeggtoxin-V on breast cancer cell line MDA-MB-231 cells can be summarized in Figure 10.

## 4. Conclusions

The gene of Latroeggtoxin-V, a new proteinaceous toxin that was found for the first time by mining the transcriptome data of *L. tredecimguttatus* eggs with the help of bioinformatics, was successfully cloned and heterologously expressed in *E. coli.* The rLatroeggtoxin-V was demonstrated to be a Na^+^/K^+^-ATPase inhibitor and could selectively act on the breast cancer cell line MDA-MB-231 cells, arresting their cell cycle, inhibiting their proliferation and migration, and inducing their apoptosis. It was speculated that the adverse effects of Latroeggtoxin-V on the breast cancer cells were exerted via affecting both of the ion transport and the receptor functions of Na^+^/K^+^-ATPase in the cells. Due to the selective and multifaceted impacts of Latroeggtoxin-V on the cancer cells, this proteinaceous toxin displays a promising application foreground in the development of anti-breast cancer drugs and the related research. The present work has also provided a new transcriptomics and bioinformatics-directed paradigm for exploring the proteinaceous toxins with too low abundance to be isolated from the natural materials.

## 5. Materials and Methods

### 5.1. Bioinformatic Analysis of Latroeggtoxin-V

Some bioinformatics online software was used to analyze the Latroeggtoxin-V. The molecular weight (MW) and isoelectric point (pI) were analyzed by compute pI/MW tool of ExPASy (http://www.expasy.org/). The conserved domain and homologous sequence of Latroeggtoxin-V was searched by the CCD database (https://www.ncbi.nlm.nih.gov/cdd/) and BLASTP program (https: //blast.ncbi.nlm. nih.gov/Blast.cgi) in NCBI. PSIPRED (http://bioinf.cs.ucl.ac.uk/psipred/) was used to analyze the secondary structure, and on this basis, the hydrophobic property and coiled-coil of α-helix in Latroeggtoxin-V were analyzed by Heliquest (http://heliquest.ipmc.cnrs.fr/) and COIL Server (https://embnet.vital-it.ch/software/COILS_form.html).

### 5.2. Gene Cloning of Latroeggtoxin-V

Trizol reagent (Invitrogen^TM^, Eugene, OR, USA) was used to extract the total RNA of the spider eggs 1–2 weeks before hatching. The purity, integrity and concentration of the extracted total RNA were detected by agarose gel electrophoresis and biophotometer (Eppendorf, Hamburg, Germany), respectively. According to the instructions in the manual of Superscript^TM^III kit (Invitrogen^TM^, Eugene, OR, USA), the total RNA was reversely transcripted to cDNA. Based on the Latroeggtoxin-V gene sequence obtained by the transcriptomic analysis of the eggs, a pair of gene-specific amplification primers were designed (Table 2). The prepared cDNA is used as the template for PCR amplification of Latroeggtoxin-V gene with the following program: initial denaturation (94 °C for 3 min), 30 cycles including denaturation (94 °C for 30 s), annealing (55 °C for 30 s), elongation (72 °C for 2 min), and final elongation (72 °C for 5 min). The reaction system is 50 μL, including 3 μL cDNA, 5 μL 10× PCR buffer, 1 μL 10 mM dNTP Mix, 2 μL F primer, 2 μL R primer, 0.5 μL *pfu*DNA polymerase (TIANGEN, Beijing, China) and 36.5 μL ddH_2_O. The PCR products were gel-purified using the QIAquick^®^ Gel Extraction Kit (Qiagen, Duesseldorf, Germany) and ligated into the pMD19-T vector after being added with a poly (A) tail. The ligation products (named pMD19-T-*Latroeggtoxin-V*) were transformed into DH5α competent cells, and the transformed cells were spread on a LB solid plate containing 100 μg/mL ampicillin. The next day, the colonies growing on the plate were picked for colony PCR detection. The positive colonies were sequenced by sequencing company to identify which contained the correct sequence.

### 5.3. Prokaryotic Expression of Latroeggtoxin-V

The pMD19-T-*Latroeggtoxin-V* plasmids were extracted by a plasmid extraction kit (TIANGEN, Beijing, China) from correctly sequenced bacteria, and then the plasmids and pET-28a expression vectors were simultaneously digested with restriction endonuclease FastDigest BamHI and FastDigest XhoI (Thermo Fisher Scientific, Waltham, MA, USA). After gel-purification and ligation of the digested products with T4 ligase (Thermo Fisher Scientific, Waltham, MA, USA), the constructed recombinant vectors pET-28a-*Latroeggtoxin-V* were transformed into BL21 (DE3). The transformed strains were plated on LB solid medium containing 100 μg/mL Kanamycin, and single colonies was selected for colony PCR detection after culturing at 37 °C for 16 h. The plasmids extracted from the colonies with positive colony PCR results were analyzed by double enzyme digestion and sequencing, and the strains with the correct analysis results were inoculated into the LB liquid medium containing 100 μg/mL Kanamycin and shaken at 37 °C and 225 r/min overnight. The next day, the bacterial solution was inoculated into 50 mL of new LB liquid medium at a ratio of 1:50, which was shaken at 37 °C and 225 r/min until the OD_600_ value reached 0.6–0.8. Prokaryotic expression was induced by the addition of IPTG (Isopropyl β-d-1-thiogalactopyranoside) to a final concentration of 0.25 mM. After 5 h of inducing incubation, SDS-PAGE was performed with a 12% SDS-polyacrylamide slab gel to detect the induced expression of Latroeggtoxin-V fusion protein.

### 5.4. Purification of Latroeggtoxin-V Fusion Protein

After expanding culture and inducing expression, the bacterial culture was centrifuged at 6000 g for 5 min to collect the precipitate, which was then resuspended in a native lysis buffer (50 mM NaH_2_PO_4_, 300 mM NaCl, 50 mM imidazole, pH8.0). The bacterial suspension was treated with the Ultrasonic crusher JY99-IIDN (SCIENTZ, Ningbo, China), and then centrifuged at 10,000 g for 30 min to collect the supernatant. For purifying Latroeggtoxin-V fusion protein, the supernatant was made to pass through a nickel-NTA agarose column (Sangon Biotech, Shanghai, China) pre-equilibrated with the native lysis buffer. The non-target proteins binding to the nickel-NTA agarose column were washed sequentially using 20 mL native lysis buffer and washing buffer (50 mM NaH_2_PO_4_, 300 mM NaCl, 100 mM imidazole, pH8.0). Latroeggtoxin-V fusion protein with His-tag was eluted with 40 mL elution buffer (50 mM NaH_2_PO_4_, 300 mM NaCl, 250 mM imidazole, pH8.0), followed by desalination and concentration with an ultrafiltration tube Millipore UFC901096 (Millipore UFC901096, Billerica, MA, USA). SDS-PAGE was used to detect the purity of the prepared Latroeggtoxin-V fusion protein sample.

### 5.5. Cleavage of Latroeggtoxin-V Fusion Protein and Isolation of rLatroeggtoxin-V

The Latroeggtoxin-V fusion protein was cleaved by enterokinase at 25 °C overnight in a cleavage buffer (20 mM Tris-HCl, 50 mM NaCl, 2 mM CaCl, pH8.0) at an enzyme: substrate ratio of 1:50 (Sangon Biotech, Shanghai, China). The cleaved products were separated with RP-HPLC (SHIMADZU, Kyoto, Japan) using a C18 column (4.6 mm × 250 mm, 5 μm particle size, Welch Xtimate^TM^, Shanghai, China). The cleaved products were eluted with a linear gradient of acetonitrile containing 0.1% TFA from 0% to 70% in 40 min at a flow rate of 1.0 mL/min. The molecular weight of the components in each elution peak was determined by electrospray ionization mass spectrometry (Waters ACQUITY UPLC/Xevo G2 QTOF, Waters, Milford, MA, USA) and thus the peak containing recombinant Latroeggtoxin-V (rLatroeggtoxin-V) was selected and lyophilized.

### 5.6. Bioactivity Assessment of rLatroeggtoxin-V against MDA-MB-231 Cells

#### 5.6.1. Cell Viability-Inhibiting Activity

Human embryonic kidney 293 (HEK293) cells, non-tumor-derived cells used as control, and breast cancer cell line MDA-MB-231 cells (Shanghai cell bank of the Chinese Academy of Sciences, Shanghai, China) were cultured in DMEM/high glucose medium (Gibco^TM^, Carlsbad, MA, USA) containing 10% fetal bovine serum (SERANA, Brandenburg, Germany) at 37 °C, 5% CO_2_ and a relative humidity of 90%, using a carbon dioxide incubator (Thermo Fisher Scientific, Waltham, MA, USA). When the cells were cultured to exponential phase, they were dissociated with trypsin digestion (Hyclone^TM^, Logan, UT, USA), collected and then seeded in a 96 well plate at a concentration of 10^3^ cells/well. After incubation for 24 h, the medium was removed by aspiration and replaced with 100 μL of experimental medium containing 5 different concentrations of rLatroeggtoxin-V (0, 20, 40, 60 and 80 μM), each of which was plated into 4 wells. After the toxin treatment was performed for 48 h, the cell viability was assessed using the 3-(4,5-dimethylthiazol-2-yl)-2,5-diphenyltetrazolium bromide (MTT) assay (Byotime, Jiangsu, China). Ten μL of MTT reagent was added to each well and the plates were incubated in the dark for 4 h at 37 °C. Subsequently, the absorbance of MTT was measured at 490 nm by a Varioska Flash plate reader (Thermo Fisher Scientific, Waltham, MA, USA). The relative cell viability was calculated using the following equation: A_test_/A_control_ × 100%, where ‘A_control_’ is the absorbance of the control (0 μM rLatroeggtoxin-V treatment) and ‘A_test_’ is the absorbance of the tests.

#### 5.6.2. Apoptosis-Inducing Activity

Hoechst 33258 staining strategy was used to observe the apoptotic morphology of the cells treated with rLatroeggtoxin-V. MDA-MB-231 cells were seeded in the 6 well plate (10^6^ cells/well), and divided into two groups: Control groups (0 μM rLatroeggtoxin-V treatment) and test groups of (40 μM rLatroeggtoxin-V treatment). After treatment for 48 h, the cells were fixed with 4% formaldehyde for 10 min and washed in PBS twice. The cells were then stained with 0.5 mL of Hoechst 33258 (Beyotime, Jiangsu, China) for 5 min, washed in PBS twice, and photographed with an Olympus IX83 fluorescence microscopy (Olympus, Tokyo, Japan) at 360 nm.

#### 5.6.3. Cell Migration-Inhibiting Activity

Wound-healing assay was used to assess the effect of rLatroeggtoxin-V on MDA-MB-231 cell migration in culture. A 24-well plate was seeded at 10^5^ cells/well and incubated until 100% confluence was reached. The layer of cells was scratched with a 100-μL pipette tip and washed with PBS three times to remove the detached cells. The incubation was continued for 24 h in fresh serum-free DMEM medium containing 4 different concentrations of rLatroeggtoxin-V (0, 20, 40 and 80 μM), each of which was plated into 3 wells. The wound widths at 0 and 24 h after scratching were measured and photographed, respectively, with a DMLB2 microscopy (LEICA, Solms, Germany) to determine the effect of rLatroeggtoxin-V on the cell migration.

#### 5.6.4. Cell Cycle Arrest Activity

The effect of rLatroeggtoxin-V on the cell cycle of MDA-MB-231 cells was assayed according to the method of Sun et al. [49]. Briefly, MDA-MB-231 cells were inoculated in 6 well plates at a concentration of 10^6^ cells /mL. After adhering to the wall, the cells were cultured in serum-free medium for 12 h so that they could grow synchronously. Different concentrations of rLatroeggtoxin-V (0, 25, 50 and 100 μM) were used to treat the cells for 48 h, and then the cells were harvested, washed in cold PBS and fixed in 75% alcohol at 4 °C for 12 h. The fixed cells were resuspended in PBS containing 0.25 mg/mL RNase A and 0.05 mg/mL propidium iodide (PI) for 30 min, and analyzed using flow cytometry (Cytoflex, Beckman, Indianapolis, IN, USA).

#### 5.6.5. Na^+^/K^+^-ATPase-Inhibiting Activity

The activity of the Na^+^/K^+^-ATPase in MDA-MB-231 cells was assayed as described [50,51,52]. MDA-MB-231 cells were seeded in 6-well plates at a density of 10^6^ cells/well in 1.8 mL volume and cultured overnight. Next day, the medium was removed by aspiration and replaced with 1.8 mL experimental medium containing 4 different concentrations of rLatroeggtoxin-V (0, 10, 100 and 1000 μM), each of which was plated into 3 wells. After treatment for 12 h, the cells were harvested by trypsinization with 0.05% trypsin and suspended in a PBS buffer. The cells were disrupted by ultrasound (Power 20%, time 3 s, interval 10 s, repeat 30 times), and the supernatant containing Na^+^/K^+^-ATPase was collected by 8000 g centrifugation at 4 °C for 10 min. The Na^+^/K^+^-ATPase activity was evaluated with a Na^+^-K^+^-ATPase detection kit (Solarbio, Shanghai, China) according to the instructions of the manufacturer. The concentration of inorganic orthophosphate (P_i_) liberated from the hydrolysis of ATP was spectrophotometrically measured at 660 nm. The amount of Na^+^/K^+^-ATPase decomposing ATP to produce 1 micromole P_i_ per milligram of cell protein per hour is one enzyme activity unit. The protein concentration was determined by the BCA (bicinchoninic acid assay) method (Beyotime, Jiangsu, China) with human serum albumin as a standard.

### 5.7. Statistical Analysis

Data were expressed as mean ± SD. Experimental data were analyzed using SPSS v19.0 statistical software (IBM Corp., Armonk, NY, USA, 2012) with the least significant differences between samples being *p* < 0.05.

## Figures and Tables

**Table 1:** Cell cycle distribution of MDA-MB-231cells treated with rLatroeggtoxin-V.

Treatment	Cell Cycle Distribution (%)		
	G_0_/G_1_	S	G_2_/M
Control (0 μM)	80.80 ± 0.69	16.22 ± 1.22	2.98 ± 0.7
25 μM	81.59 ± 1.65	15.81 ± 1.91	2.98 ± 0.7
50 μM	83.25 ± 0.73 ^a^	13.83 ± 0.77 ^a^	2.91 ± 0.15
100 μM	89.67 ± 1.39 ^a^	8.33 ± 0.38 ^a^	2.00 ± 1.09

^a^ significantly different compared with the control (*p* < 0.05). Each group of data comes from the average of three measurements, expressed as mean ± SD. (*n* = 3).

**Table 2:** Primer sequences.

Primer Name	Primer Sequence (5′→3′)
*Latroeggtoxin-V*-F	CATGGGATCC$\underset{=}{\mathrm{GACGACGACGACAAG}}$TCTCAGGCTGGTGAATGGGGTTCTG
*Latroeggtoxin-V*-R	CCGCTCGAGTTAACCGTCGATTTTTTTTTCTTCTTTAGC

Notes: The single underline indicates restriction endonuclease cleavage sites, whereas the double underline indicates enterokinase cleavage site. GGATCC and CTCGAG are cleavage sites of BamHI and XhoI, respectively.
